# Association Between Depression, Health Beliefs, and Face Mask Use During the COVID-19 Pandemic

**DOI:** 10.3389/fpsyt.2020.571179

**Published:** 2020-10-22

**Authors:** Daniel Thomas Bressington, Teris Cheuk Chi Cheung, Simon Ching Lam, Lorna Kwai Ping Suen, Tommy Kwan Hin Fong, Hilda Sze Wing Ho, Yu-Tao Xiang

**Affiliations:** ^1^School of Nursing, The Hong Kong Polytechnic University, Kowloon, Hong Kong; ^2^College of Nursing and Midwifery, Charles Darwin University, Casuarina, NT, Australia; ^3^Squina International Center for Infection Control, The Hong Kong Polytechnic University, Kowloon, Hong Kong; ^4^Department of Psychology, York University, Toronto, ON, Canada; ^5^Faculty of Health Sciences, University of Macau, Macau, China

**Keywords:** depression, health belief model, face mask, COVID-19, Hong Kong

## Abstract

The 2019 novel coronavirus (COVID-19) pandemic is associated with increases in psychiatric morbidity, including depression. It is unclear if people with depressive symptoms understand or apply COVID-19 information differently to the general population. Therefore, this study aimed to examine associations between depression, health beliefs, and face mask use during the COVID-19 pandemic among the general population in Hong Kong. This study gathered data from 11,072 Hong Kong adults via an online survey. Respondents self-reported their demographic characteristics, depressive symptoms (PHQ-9), face mask use, and health beliefs about COVID-19. Hierarchical logistic regression was used to identify independent variables associated with depression. The point-prevalence of probable depression was 46.5% (*n* = 5,150). Respondents reporting higher mask reuse (*OR* = 1.24, 95%CI 1.17–1.34), wearing masks for self-protection (*OR* = 1.03 95%CI 1.01–1.06), perceived high susceptibility (*OR* = 1.15, 95%CI 1.09–1.23), and high severity (*OR* = 1.33, 95%CI 1.28–1.37) were more likely to report depression. Depression was less likely in those with higher scores for cues to action (*OR* = 0.82, 95%CI 0.80–0.84), knowledge of COVID-19 (*OR* = 0.95, 95%CI 0.91–0.99), and self-efficacy to wear mask properly (*OR* = 0.90 95%CI 0.83–0.98). We identified a high point-prevalence of probable major depression and suicidal ideation during the COVID-19 outbreak in Hong Kong, but this should be viewed with caution due to the convenience sampling method employed. Future studies should recruit a representative probability sample in order to draw more reliable conclusions. The findings highlight that COVID-19 health information may be a protective factor of probable depression and suicidal ideation during the pandemic. Accurate and up-to-date health information should be disseminated to distressed and vulnerable subpopulations, perhaps using digital health technology, and social media platforms to prompt professional help-seeking behavior.

## Background

The novel coronavirus (2019-nCoV) has been transmitting around the world since January 2020. The resulting COVID-19 pandemic has undoubtedly resulted in great medical and psychosocial challenges that can damage mental health, including potentially increasing rates of depression.

Depression is a common mental disorder that is highly prevalent in the general population and is a major contributor to the overall global burden of disease (1). The importance of depression worldwide is illustrated by its inclusion as a priority condition within the World Health Organization's Mental Health Gap Action Programme (2). The average point prevalence of depression in the absence of a global pandemic has been recently reported to be 12.9% across 30 countries (3). However, preliminary evidence highlights that levels of stress, fear, anxiety, Post-traumatic stress disorder (PTSD), sleep disorders and depressive symptoms may dramatically increase in response to the COVID-19 pandemic (4–6). It is also possible that suicide rates may increase due to a variety of COVID-19 related issues, such as financial hardship, loneliness and lack of support (7).

A number of studies have been published reporting the mental health impact of the COVID-19 pandemic, but the majority of studies on the prevalence of depressive symptoms during COVID-19 have been conducted in mainland China and are not directly generalizable to settings with lower rates of infections and deaths. These internet-based surveys report varied depression prevalence rates in the general Chinese population, for example, 17.1% (6), 20.1% (8), and 34.7% (9). However, direct comparisons of prevalence estimates from these studies are impossible due to the use of different screening and diagnostic approaches and the inclusion of different subpopulations. Despite these complications, interestingly, one study involving 205 participants (9) found lower rates of probable major depression in people who had been infected by the virus (29.2%) and in those who had been officially quarantined (9.8%), when compared to the general public (34.7%). This may suggest that the fear of infection within the context of social restrictions is more psychologically challenging than actually contracting the disease or being subjected to enforced quarantine measures.

At the time of writing (late May 2020), the numbers of COVID-19 infections in Hong Kong were lower than many other countries, with just over 1,066 known infections and four confirmed deaths. Despite these comparatively low infection rates, the Hong Kong public may also be experiencing an increase in depressive symptoms as people have been experiencing the continuous fear of COVID-19 and restrictions on their daily lives since mid-January 2020. Still, it is currently unclear how this prolonged psychosocial stress has impacted on mental health because information on the rates of depressive symptoms in Hong Kong during COVID-19 is scarce. A recent cross-sectional survey highlights the possibility of increased anxiety; 88% of over 1,000 Hong Kong citizens reported a high perceived susceptibility of being infected with COVID-19 and the mean anxiety level of 8.82 was borderline abnormal as measured by the Hospital Anxiety and Depression Scale (10). Also, a large internet survey (11) with over 52,000 responses from 36 regions of China, including the Special Administrative Regions of Hong Kong and Macao, reported that overall 35% of respondents were experiencing COVID-19 related psychological distress. The highest rates of distress were found in the central area of China, which includes Hubei province where the virus was first detected, perhaps suggesting that regions of China with lower infection rates, such as Hong Kong, may experience a lesser impact of COVID-19 on mental health (11). The current lack of empirical evidence on depression rates in Hong Kong during COVID-19 is an important gap in understanding because such information would help to inform mental health service planning and the development of policies to promote mental health in the community.

It is also important to better understand how people with depressive symptoms may perceive the severity of COVID-19 and their susceptibility to being infected as this could influence how they respond to, and comply with public health advice and policies designed to reduce infection rates. Given that self-care and other health behaviors are often sub-optimal in people with depression with chronic physical illnesses (12, 13), it is logical to assume that similar issues may exist in infection control behaviors. Indeed, poor adherence to health behavior advice in people with depression is in part due to cognitive, motivational, and volitional deficits associated with the illness, such as poor self-efficacy and negative outcome expectations (14). In Hong Kong, the public is advised to adopt a range of measures to prevent virus transmission, consisting of maintaining a safe distance from others, performing good hand hygiene, and wearing face masks when in public (15). There is currently conflicting advice about the use of personal protective equipment (PPE), such as face masks, across different countries and from the WHO (16). However, wearing a surgical mask when unwell has become very common in Hong Kong since the outbreak of the COVID-19 pandemic, with a recent survey reporting that 98.8% of 1,005 people in Hong Kong wore face masks when venturing outside their homes (17).

Despite the popularity of face masks and the Hong Kong government's advice to wear a mask in certain situations (15), it is currently unknown if safe guidelines for use are adhered to or clearly understood, particularly amongst people with depressive symptoms. Furthermore, with the limited supply of face masks, the practice of reusing face masks has not been explored. The limited earlier studies on the use of PPE and safety practices in people who are depressed have mainly involved farmers. These studies reported that farmers with depressive symptoms in the USA were more likely to engage in high-risk safety behaviors most associated with farm injuries than those without depressive symptoms (18) and that low levels of safety knowledge in depressed individuals were more strongly associated with injuries than in those without depressive symptoms (19). Therefore, research on how depressive symptoms are associated with infection prevention behaviors and COVID-19 related health beliefs is imperative, particularly due to the apparent recent increases in psychological distress within the general population. In order to reduce the potential of confounding factors associated with age (i.e., proven susceptibility to severe complications from COVID-19 or age-related capacity to complete the survey) and to enhance direct comparability with previously published studies, we included only working aged adults (aged 18–59 years) in the current study.

The Health Belief Model (HBM) (20) was adopted as a general conceptual framework to hypothesize that bidirectional relationships may exist between participants' level of depressive symptoms, their COVID-19 related beliefs and mask wearing practice. We tentatively hypothesized that COVID-19 related health beliefs and infection control behaviors induced by the pandemic would exacerbate transient or pre-existing chronic depressive symptoms (possibly because people may feel overwhelmed by the perceived risk of COVID-19 infection, but perceive they are ill-equipped to protect themselves) (21). Subsequently, the resulting cognitive distortions/deficits associated with increases in depressive symptoms [i.e., perceived poor self-efficacy and negative outcome expectations (14)] may further trigger and maintain depressive symptoms. Although it is impossible to demonstrate temporal relationships due to the cross-sectional nature of the current study, we hoped to obtain preliminary evidence that people who are depressed may conceptualize, understand, and act upon COVID-19 related health beliefs differently than those with low levels of depressive symptoms. Such information would have implications for the design and delivery of targeted COVID-19 public health information. The findings could also be used by mental health professionals to profile typical COVID-19 related health beliefs and face mask use patterns in people who are being treated for depression in order to devise empowering psychoeducational interventions with the potential to enhance self-efficacy, improve safety of face mask use, and thus reduce levels of distress that maintain depression.

Given the aforementioned knowledge gaps and general study aims, the specific objectives of this study were to: (a) establish the point prevalence of depressive symptoms in working-aged adults in the general Hong Kong population and; (b) profile and compare COVID-19 related health beliefs and face mask use in individuals with and without depressive symptoms.

## Methods

### Study Design and Setting

This large internet-based cross-sectional study was conducted in the general population in Hong Kong during the outbreak of COVID-19 using a convenience sampling method.

### Participants and Inclusion/Exclusion Criteria

To be eligible, participants needed to be Hong Kong working-aged residents, aged 18–59 years and able to read English or Chinese.

### Recruitment of Subjects/Data Collection

The questionnaire was delivered to several online platforms (i.e., Google form and Qualtrics), including a discussion forum, community peer groups (e.g., COVID-19 information group, child parenting group, working adult peer groups, etc.), and organizational or personal Facebook pages. The subject line of the invitation was: Study about face mask use among the general public during COVID-19 (Hong Kong). Data collection spanned from 24 March to 20 April 2020. Given that this was a self-selecting sample, we aimed to recruit as many participants as possible over the recruitment period to improve the potential representativeness of the sample, and thus did not calculate a minimum sample size a-priori.

### Ethical Considerations

This study was approved by the Human Subjects Ethics Sub-committee of the Hong Kong Polytechnic University (reference no: HSEARS20200227002-01). Participants provided their written informed consent prior to participation online. Participants were assured of their anonymity and confidentiality, and their rights of withdrawal were respected. Given the sensitive nature of some of the questions, and the potential for some respondents to experience distress when considering their mood/suicidal ideation, we provided contact details where they could receive a referral for professional emotional support and receive additional advice.

### Instruments

Participants were required to fill in a questionnaire (presented in bilingual mode: Traditional Chinese and English languages) comprising four sections. Section A solicited information regarding participants' gender, age, marital status, educational level, occupation, monthly household income, whether they have direct patient contact (yes/no), and the frequency of experiencing influenza like symptoms in the past 12 months. All questionnaires are available from the corresponding author upon request.

Section B included the face mask use scale (FMUS) (22) which involved two categories: (1) protect self, (2) protect others; and in three areas: (1) public, (2) clinic, (3) home. The relevant mask types were clearly defined at the start of the questionnaire (i.e., paper/gauze, washable sponge/cotton, surgical, activated carbon, and N95 respirator). This scale comprised 6 items on a 5-point scale indicating the frequency of face mask use practice. Scores ranged from 0 to 24 representing the overall practice of FMU. Higher score indicated higher frequency of FMU. The psychometric properties of the Chinese version of the FMUS were satisfactory, with Cronbach's alpha of 0.80–0.81 and the corrected item-total correlation coefficients of 0.46~0.67. The test-retest stability of intraclass correlation coefficient was *r* = 0.84 (23).

Section C solicited participants' understanding of the COVID-19 public health risk and their reasons for face mask use. Thirteen questions were asked to examine the HBM components in participants. These included perceived susceptibility toward the COVID-19 outbreak, the severity of the pandemic, cues to action for self-protection by the government /family members/friends, perceived benefits/barriers of wearing masks, their knowledge of COVID-19 and the self-efficacy of wearing a mask properly. All the questions constructed in this section were derived from the Health Belief Model (HBM), which was used as a conceptual framework to explain health-related behaviors on face mask use. The HBM is most widely used framework for predicting and examining face mask use in previous studies (24–26) and the components of Health Belief Model were shown to be the significant factors in explaining face mask use (26). These items were translated into Chinese based on the principles of Brislin's model of forward and backward translation (27). The items were then revised to enhance the relevance. A panel of six experts evaluated the relevance of these items for measuring the said concepts and a satisfactory content validity of all items was obtained. Participants indicated their response on a 4-point scale (1: not at all; 2: slightly; 3: very; 4: extremely). Higher scores indicated that participants were highly aware of the public health risk brought by COVID-19 and also reflected their face mask use patterns. Examples of questions (and the associated HBM component) include: Do you feel vulnerable to contracting the disease (perceived susceptibility)? What is the degree to which you are worried that your living place would become a quarantine city because of the widespread outbreak of the disease in the community (perceived severity)? What is the degree to which you agree wearing facemasks could prevent contracting and spreading the disease (perceived benefits)? What is the degree to which you have difficulty in obtaining facemasks (perceived barriers)? What is the degree to which the local government encouraged you to wear facemasks (cues to action)? What is the degree to which you believed you were able to properly wear face masks (self-efficacy)?

Section D assessed participants' depressive symptoms using the PHQ-9. This measure consists of nine items to measure the presence and severity of self-reported depressive symptoms in the previous 2 weeks. Each item ranges from 0 to 3, with a summed total score ranged from 0 to 27. A score of 5–9 indicated ‘mild’ depressive symptoms, 10–14 ‘moderate’ depressive symptoms, 15–19 ‘moderately severe’ depressive symptoms and ≥ 20 ‘severe’ depressive symptoms. In accordance with established procedures, participants with a total PHQ9 score of ≥10 were classified as having probable depression. Cronbach's alpha for the internal consistency reliability of the Chinese version of the PHQ-9 was 0.86 and the correlation coefficient for the 2-week test–retest of the total score was 0.86 (28). The Cronbach Alpha for PHQ-9 in this study was 0.91. The Chinese version of the PHQ9 was validated by comparing its scores with the clinical diagnosis of a major depressive episode, using the DSM-IV criteria (AUC = 0.95, sensitivity = 0.88, specificity = 0.88) at the cut-off point of 9/10 with good internal consistency (Cronbach's α = 0.89) (29).

### Statistical Analysis

Data analyses were performed using SPSS 25.0 for Windows (SPSS Inc., Chicago, IL, USA). Descriptive analysis, chi-square statistics and independent samples *t*-tests were used to examine the associations between sociodemographic characteristics, face mask use, core components of health belief model and depression. Hierarchical logistic regression analysis was performed to identify factors which were independently associated with depressive symptoms, in order to test our tentative hypothesis that COVID-19 related health beliefs and face mask use patterns/beliefs would account for a significant amount of variance in depressive symptoms. The total score of the PHQ-9 was the dependent variable, with a cut-off point of ≥10 indicating probable depression. All the significant sociodemographic characteristics, face mask use patterns, and HBM components were entered in the multivariate binary logistic regression analysis as independent variables in a hierarchical procedure. The level of significance was set as *p* < 0.05 (two-tailed).

## Results

A total of 11,072 participants fully completed the online survey (52.5% of those who started the survey). Due to the nature of recruitment/sampling and the online survey mode, we are unable to calculate a survey response rate. We excluded around 300 responses that were ineligible to participate due to their age (i.e., over 59 and under 18 years). Table 1 reports the severity of depressive symptoms and response to the suicidality/self-harm ideation question for the entire sample and across genders. A disproportionate number (*n* = 8,815, 80.7%) were female. Participants' age ranged from 18 and 59 years, with those aged 31 and 40 being most represented (20% of the entire sample). Over two-thirds (68.3%, *n* = 7,466) were married. Participants were generally well-educated, with less than one quarter of (24.6%) only having obtained secondary school education or below. Around one in 10 (*n* = 1,217, 11%) were health professionals. Most respondents (38.4%, *n* = 4,257) earned 5,130 USD or less per month. There were small statistically significant differences in demographic characteristics across males/female groups (all *ps* < 0.05), for example in relation to age group distribution, marital status, education level and occupation (please see Table 2). These significant differences may suggest that that the results may not be generalisable to both genders.

**Table 1:** Prevalence/severity of depressive symptoms and suicide/self-harm ideation.

	**Entire sample**	**Male**	**Female**	**Chi-square/*t*-test (df)**	***P*-value**
**PHQ-9 total** Mean (*SD*)	9.60 (6.04)	9.71 (6.24)	9.58 (6.00)	*t* (0.88) (10918)	0.38
**Depression severity** *n* (valid %)	11,072 (100)	2105 (19.3)^#^	8815 (80.7)^#^	4.88 (4)	0.30
Minimal/None	2,463 (22.2)	484 (23.0)	1945 (22.1)		
Mild	3,459 (31.2)	628 (29.8)	2788 (31.6)		
Moderate	2,820 (25.5)	529 (25.1)	2246 (25.5)		
Moderately severe	1,609 (14.5)	311 (14.8)	1278 (14.5)		
Severe	721 (6.5)	153 (7.3)	558 (6.3)		
**Probable depression** *n* (valid %)	5,150 (46.5)	993 (47.17)	4082 (46.31)	0.51 (1)	0.47
**Suicide/self-harm ideation** *Thoughts that you would be better off dead, or of hurting yourself in some way*				25.34 (3)	<0.001^***^
Not at all	8,584 (77.5)	1547 (73.5)	6922 (78.5)		
Several days	1,711 (15.5)	387 (18.4)	1300 (14.8)		
More than half	567 (5.1)	121 (5.7)	435 (4.9)		
Nearly everyday	210 (1.9)	50 (2.4)	158 (1.8)		

Cut off points for PHQ-9: Score 0–4 “minimal/none”; 5–9 “mild”; 10–14 “moderate”; 15–19 “moderately severe”; 20–27 “severe.”

*Probable depression (PHQ-9 score ≥ 10)*.

# Missing value (1.4%, n = 152)

*** *p < 0.001*.

**Table 2:** Demographic characteristics, face mask use and health beliefs by genders.

	**Entire sample (*****n*** **=** **10,920)**		**Male (*****n*** **=** **2,105)**		**Female (*****n*** **=** **8,815)**		**χ^2^/*t* (d.f.)**	***p*-value**
	***N***	**valid%**	***N***	**%**	***N***	**%**		
**Age (years)**								
18–30	2,025	19.11	458	22.34	1567	18.33	17.57 (3)	0.001^**^
31–40	4,342	40.97	801	39.07	3541	41.43		
41–50	3,161	29.83	585	28.54	2576	30.14		
51–59	1,069	10.09	206	10.05	863	10.10		
**Marital status**								
Single	3,073	28.27	591	28.17	2482	28.29	12.38 (2)	0.002^**^
Married/In a relationship	7,438	68.41	1463	69.73	5975	68.10		
Divorced/Separated/Widowed	361	3.32	44	2.10	317	3.61		
**Education level**								
Elementary or below	25	0.23	7	0.33	18	0.21	33.18 (2)	<0.001^***^
High School	2,651	24.36	410	19.57	2241	26.03		
University or higher	8,208	75.41	1678	80.10	6350	73.76		
**Occupation**								
Healthcare workers	1,214	11.15	162	7.72	1052	11.96	30.78 (1)	<0.001^***^
Non-healthcare workers	9,677	88.85	1936	92.28	7741	88.04		
**Monthly income (USD)**								
<2,650	3,913	36.26	575	27.56	3338	38.35	96.46 (3)	<0.001^***^
$2,651–5130	4,236	39.25	895	42.91	3341	38.38		
$5,131–7,700	1,671	15.49	364	17.45	1307	15.01		
≥7,701	971	9.00	252	12.08	719	8.26		
**Experiencing influenza-like symptoms in the past year**								
No	4,231	55.23	833	58.05	3398	54.59	5.65 (1)	0.018^*^
Yes	3,429	44.77	602	41.95	2827	45.41		
**Safety of reusing face mask**								
Very unsafe	3,722	34.08	658	31.26	3,064	34.76	27.24 (4)	<0.001^***^
Unsafe	3,898	35.70	745	35.39	3,153	35.77		
Unsure	2,241	20.52	440	20.90	1,801	20.43		
Safe	1,018	9.32	250	11.88	768	8.71		
Very safe	41	0.38	12	0.57	29	0.33		
**Transparency of face mask reuse guidelines**								
Very unclear	3,432	31.45	797	37.88	2,635	29.91	65.95 (3)	<0.001^***^
Unclear	5,251	48.12	969	46.06	4,282	48.61		
Clear	2,020	18.51	294	13.97	1,726	19.59		
Very clear	210	1.92	44	2.09	166	1.88		
	**Mean**	***SD***	**Mean**	***SD***	**Mean**	***SD***		
Frequency of reuse face mask	1.66	0.75	1.73	0.85	1.65	0.73	*t* 4.78 (10,918)	<0.001^***^
Face mask use	24.48	4.01	24.71	4.26	24.43	3.95	*t* 2.95 (10,918)	0.003^**^
Subscale of self-protection	11.98	2.04	12.20	2.16	11.93	2.01	*t* 5.37 (10,918)	<0.001^***^
Subscale of protecting others	12.50	2.34	12.52	2.45	12.49	2.31	*t* 0.39 (10,918)	0.70
Susceptibility for infection	2.95	0.89	2.88	0.89	2.96	0.89	*t* −3.98 (10,865)	<0.001^***^
Severity after infection	6.56	1.41	6.41	1.46	6.60	1.39	*t* −5.58 (10,893)	<0.001^***^
Cues to action	14.10	1.76	13.97	1.81	14.13	1.74	*t* −3.75 (10,838)	<0.001^***^
Knowledge on outbreak	5.21	1.09	5.15	1.16	5.22	1.08	*t* −2.92 (10,884)	0.003^**^
Self-efficacy using face masks	3.33	0.57	3.35	0.60	3.33	0.56	*t* 1.38 (10,904)	0.17

* *p < 0.05*,

** p < 0.01, and

*** *p < 0.001*.

*Chi-square/t-tests comparing depressed/non-depressed groups*.

In consideration of the first study objective, to establish the point prevalence of depressive symptoms in working-aged adults in the general Hong Kong population, the mean score of depression in this study was 9.06 (*SD* 6.04), indicating an overall mild level of depressive symptoms for the entire sample. A total of 46.5% of the sample reported at least a moderate level of depressive symptoms (total PHQ-9 score ≥10), suggesting a probable major depressive disorder, with no differences across genders (*p* > 0.05). A concerning proportion of the overall sample (22.5%) had suicide or self-harm ideation for at least several days over the previous 2 weeks, with more males reporting this than their female counterparts (26.5 vs. 21.5%). Significant differences were also observed in the frequencies of suicide/self-harm thoughts across genders (*p* < 0.001).

In consideration of the second study objective (to profile and compare COVID-19 related health beliefs and face mask use in individuals with and without depressive symptoms), Table 2 provides details of health beliefs/face mask use across genders and Table 3 reports the sociodemographic characteristics, face mask use, and COVID-19 health beliefs of the whole sample and the probable depression/non-depression groups. Chi-square test of independence revealed that there were statistically significant associations between probable depression and categories of age, marital status, educational level, occupation, monthly household income, experiencing influenza-like symptoms in the past year, safety of reusing face mask, and transparency of face mask reuse guidelines (all *p* < 0.05). Results from the independent samples *t*-tests showed that participants' frequency of reusing face masks, susceptibility, perceived severity, cues to action on taking precautionary measures against the infection, knowledge of the coronavirus disease outbreak and self-efficacy to wear mask properly were significantly different across the probable depression and no depression groups (all *p* < 0.005). Similarly, there were small but significant differences in COVID-19 related health beliefs and face mask use across genders (all *p* < 0.05) apart from the “protecting others” and “self-efficacy using face masks” subscales.

**Table 3:** Demographic characteristics, face mask use, and health beliefs by depression category.

	**Entire sample (*****n*** **=** **11,072)**		**Depression (*****n*** **=** **5,150)**		**No depression (*****n*** **=** **5,922)**		**χ^2^/*t* (d.f.)**	***p*-value**
	***N***	**Valid%**	***N***	**%**	***N***	**%**		
**Age (years)**								
18–30	2,028	19.1	1,182	23.8	846	14.9	311.95 (3)	<0.001^***^
31–40	4,348	40.9	2,179	44.0	2,169	38.2		
41–50	3,181	29.9	1,277	25.8	1,904	33.5		
51–59	1,079	10.1	319	6.4	760	13.4		
**Marital status**								
Single	3,092	28.3	1,570	30.9	1,522	26.1	33.29 (2)	<0.001^***^
Married/In a relationship	7,466	68.4	3,334	65.7	4,132	70.7		
Divorced/Separated/Widowed	362	3.3	174	3.4	188	3.2		
**Education level**								
Elementary or below	25	0.2	9	0.2	16	0.3	7.05 (2)	0.03^*^
High School	2,668	24.4	1,186	23.3	1,482	25.3		
University or higher	8,244	75.4	3,888	76.5	4,356	74.4		
**Occupation**								
Healthcare workers	1,217	11.1	520	10.2	697	11.9	7.95 (1)	0.005^**^
Non-healthcare workers	9,731	88.9	4,574	89.8	5,157	88.1		
**Monthly income (USD)**								
<2,650	3,929	36.2	1,966	38.9	1,963	33.9	64.75 (3)	<0.001^***^
$2,651–5,130	4,257	39.3	2,015	39.9	2,242	38.7		
$5,131–7,700	1,678	15.5	704	13.9	974	16.8		
≥7701	977	9.0	369	7.3	608	10.5		
**Experiencing influenza-like symptoms in the past year**								
No	4,284	55.2	1,837	50.77	2,447	59.0	53.31 (1)	<0.001^***^
Yes	3,479	44.8	1,781	49.23	1,698	41.0		
**Safety of reusing face mask**								
Very unsafe	3,777	34.1	1,828	35.5	1,949	32.9	73.70 (4)	<0.001^***^
Unsafe	3,957	35.7	1,890	36.7	2,067	34.9		
Unsure	2,262	20.4	1,064	20.7	1,198	20.2		
Safe	1,035	9.3	354	6.9	681	11.5		
Very safe	41	0.4	14	0.3	27	0.5		
**Transparency of face mask reuse guidelines**								
Very unclear	3,470	31.4	1,776	34.6	1,694	28.6	79.13 (3)	<0.001^***^
Unclear	5,312	48.1	2,470	48.1	2,842	48.0		
Clear	2,060	18.6	819	15.9	1,241	21.0		
Very clear	213	1.9	75	1.5	138	2.4		
	**Mean**	***SD***	**Mean**	***SD***	**Mean**	***SD***		
Frequency of reuse face mask	1.66	0.75	1.68	0.76	1.65	0.74	*t* −2.23 (11,070)	0.03^*^
Face mask use	18.48	4.00	18.46	4.10	18.51	3.90	*t* −0.53 (11,070)	0.58
Subscale of self-protection	8.98	2.04	9.01	2.00	8.96	2.07	*t* −1.22 (11,070)	0.22
Subscale of protecting others	9.50	2.34	9.50	2.25	9.50	2.41	*t* 0.11 (11,070)	0.91
Susceptibility for infection	2.95	0.90	3.07	0.89	2.84	0.88	*t* −13.96 (11,003)	<0.001^***^
Severity after infection	6.56	1.41	6.94	1.22	6.24	1.47	*t* −26.87 (11,031)	<0.001^***^
Cues to action	14.10	1.76	13.68	1.71	14.48	1.71	*t* 24.52 (10,973)	<0.001^***^
Knowledge on outbreak	5.21	1.09	5.05	1.09	5.34	1.08	*t* 13.85 (11,023)	<0.001^***^
Self-efficacy using face masks	3.33	0.57	3.29	0.57	3.37	0.57	*t* 7.89 (11,043)	<0.001^***^

* *p < 0.05*,

** p < 0.01, and

*** *p < 0.001*.

*Chi-square/t-tests comparing depressed/non-depressed groups*.

Table 4 shows the results of regression analyses using probable depression as the dependent variable. Three models were built using multivariate binary logistic regression in which independent variables were entered the final model in a hierarchical procedure in three stages. Participants' sociodemographic variables and experiencing influenza-like symptoms in the past year were entered in Model 1. In Model 2, variables from Model 1 remained in the regression analysis as control confounding variates. Variables for face mask use and COVID-19 related beliefs were also entered.

**Table 4:** Binary Logistic Regression identifying variables associated with depressive symptoms.

**Factors**	**Model 1**		**Model 2**		**Model 3**	
	**OR**	**95% CI**	**OR**	**95% CI**	**OR**	**95% CI**
Constant	3.042		2.733		5.130	
Age (range)^∧^	0.962	(0.957, 0.967)^***^	0.962	(0.957, 0.967)^***^	0.973	(0.967, 0.978)^***^
Gender (male)	1.034	(0.934, 1.144)	0.987	(0.891, 1.094)	1.033	(0.928, 1.150)
No. of persons living together (living alone)	1.016	(0.990, 1.042)	1.015	(0.989, 1.042)	1.002	(0.975, 1.030)
Close contact with patients (yes)	1.017	(0.962, 1.076)	1.009	(0.954, 1.068)	0.998	(0.941, 1.059)
Monthly income	0.943	(0.915, 0.972)^***^	0.944	(0.916, 0.973)^***^	0.964	(0.935, 0.995)^*^
Experiencing influenza-like symptoms in the past year	1.058	(1.044, 1.071)^***^	1.058	(1.045, 1.072)^***^	1.041	(1.028, 1.055)^***^
Occupation (Healthcare workers)	1.295	(1.059,1.584)	1.236	(1.082, 1.625)	1.088	(0.880, 1.345)
Education (University or above)	0.987	(0.890, 1.095)	0.989	(0.890, 1.098)	1.103	(0.988, 1.231)
Frequency of reuse face mask			1.270	(1.194, 1.352)^***^	1.243	(1.165, 1.327)^***^
Safety in reusing face mask (safe)			0.875	(0.834, 0.918)^***^	0.934	(0.888, 0.982)^**^
Transparency of face mask reuse guidelines (clear)			0.823	(0.779, 0.869)^***^	0.920	(0.869, 0.975)^**^
Face masks for self-protection			1.038	(1.010, 1.066)^**^	1.033	(1.004, 1.062)^*^
Face masks for protecting others			0.992	(0.969, 1.016)	0.985	(0.962, 1.010)
Susceptibility for infection					1.148	(1.092, 1.206)^***^
Severity after infection					1.326	(1.282, 1.371)^***^
Cue					0.821	(0.799, 0.842)^***^
Knowledge					0.951	(0.911, 0.992)^*^
Efficacy					0.903	(0.834, 0.977)^*^
Adjusted R^2^	0.057		0.072		0.164	

*OR, odds ratio; CI, confidence interval*.

* *p < 0.05*,

** p < 0.01, and

*** *p < 0.001*.

∧ *range refers to the defined age range in Tables 2, 3*.

Core elements of the HBM were entered at Model 3 along with the variables from Model 1 and 2. The adjusted *R* square was 0.164 indicating that the significant predictors identified in this final regression model accounted for 16% of the variance in depression. Results show that in terms of demographics, older participants (*OR* 0.97, 95% CI 0.97, 0.98) and those who earned a monthly household income of USD 7,701 or above (*OR* 0.96, 95% CI 0.94, 0.99) were less likely to be depressed. Whereas, participants who had experienced influenza-like symptoms in the past year were more likely to report depression (*OR* 1.04, 95% CI 1.03, 1.06).

In relation to face mask use/health beliefs, participants who had higher frequency of reusing masks (*OR* 1.24, 95% CI 1.17, 1.33), those wearing face masks for self-protection (*OR* 1.03 95% CI 1.00, 1.06), believed themselves to be more susceptible to the disease (*OR* 1.15, 95% CI 1.09, 1.21) and perceived high severity of COVID-19 illness (*OR* 1.33, 95% CI 1.28, 1.37) were more likely to report depressive symptoms. Whereas, the likelihood of having probable depression was lower in participants that reported feeling safe reusing facemasks (*OR* 0.93, 95% CI 0.89, 0.98), higher scores for cues to action (*OR* 0.82, 95% CI 0.80, 0.84), knowledge of the disease pandemic (*OR* 0.95 95% CI 0.91, 0.99), and self-efficacy to wear masks properly (*OR* 0.90 95% CI 0.83, 0.98). Participants who were unclear about mask reuse guidelines, however, were more likely to report depression than those who thought the guidelines were clear (*OR* 0.92 95% CI 0.87, 0.98).

## Discussion

The overall point-prevalence of probable depression (as defined by a total PHQ-9 score ≥10) in the 11,072 respondents was 46.5%, which is four times greater than the estimate of 11.2% in Hong Kong in late 2019 using the same cut-off score (30) and far higher than prevalence of 4.3% of respondents with PHQ9 scores >9 reported in a household telephone survey involving over 6,000 people in the Hong Kong general population (31). This is also greater than the 34% of the general population who reported PHQ9 scores of ≥10 in mainland China during COVID-19 (9). While our findings suggest higher levels of depressive symptoms than other Chinese studies, direct comparisons should be viewed with caution due to the fact that the current study was conducted during a time when people in Hong Kong were facing great adversities associated with widespread social unrest and economic concerns in conjunction with fears about the emerging pandemic. Despite these contextual differences, the current study's findings share some important characteristics with previous studies involving Chinese people, specifically that probable depression was found to be more likely in those that are younger and those in lower income brackets, a result that seems to concur with findings from a survey involving 10,000 primary care patients in Hong Kong (32) and a recent Chinese web-based survey (33) that reported rates of depression during COVID-19 were highest in people aged under 35 years.

Although the very high levels of depressive symptoms are concerning, it is possible that these reported symptoms could be artifacts of various confounding factors and methodological shortfalls. For example, due to the cross-sectional design of the study we cannot be sure that the PHQ9 data collected are specifically measuring COVID-19-related depressive symptoms because it is impossible to differentiate pre-existing depressive symptoms from those recently triggered by the COVID-19 pandemic. This is a particularly important consideration given that high levels of depressive symptoms may have already existed in the sample due to the social unrest evident in Hong Kong since 2019. It is also important to highlight that many of the 46.5% of participants with symptoms suggestive of probable depression would be unlikely to be diagnosed with major depression because the depressive symptoms may be transient and PHQ9 is a screening tool that measures severity of depressive symptoms rather than being a diagnostic instrument. Indeed, a diagnostic meta-analysis of the PHQ9 reported only reasonable diagnostic accuracy using the summed score method, with a pooled sensitivity and specificity of 0.78 [95% CI, 0.70–0.84] and 0.87 (95% CI, 0.84–0.90), respectively when using a cut off score of ≥10 (34).

Although many of the reported depressive symptoms may be transient, it is extremely concerning that 21% (*n* = 2,330) of respondents in the current study reported moderately-severe to severe depressive symptoms and 7% (*n* = 777) indicated that they had thoughts of suicide and/or self-harm on the majority of days in the previous 2 weeks. Treatment guidelines suggest that such high levels of depressive symptoms and suicidality require prompt active treatment with psychotherapy and or/medications from mental health services (32, 35). Contextually, these findings are worrying because figures from the Hong Kong Hospital Authority (36) indicate that 45,800 people, or around 1% of the working-aged adult population of 4.4 million (37) are treated annually for depression by specialist inpatient/outpatient psychiatric services. Given that 6% of people in the current study reported severe depressive symptoms warranting prompt psychiatric treatment, it is quite possible that the already stretched Hong Kong mental health services could be overwhelmed if the reported symptoms are not transient and do not subside after the pandemic resolves.

Our findings of an increase in psychiatric morbidity during COVID-19 seem to concur with research conducted in the early stages of the 2002–2003 SARS outbreak, which report increases in rates of suicidality and persistent depression (38). However, the levels of depressive symptoms in the current study were reported in the midst of a spike in the numbers of Hong Kong infections. Therefore, future studies conducted once the pandemic resolves and that utilize stratified random sampling to recruit a representative sample are urgently required to confirm the generalizability and veracity of our results.

The overall use of face masks in the current study (as indicated by the total FMUS score) is high, however similar studies are very rare and this limits opportunities to make direct comparisons. Before the COVID-19 outbreak, some local data indicated a medium total face mask use score (i.e., mean = 9.78–10.63, *SD* 4.89–5.40) among 971 members of the general public (23). Whereas, the current results (mean = 18.5, *SD* 3.90–4.10) indicate a great increase in frequency of face mask use practice since the pandemic. Furthermore, our results related to health beliefs on COVID-19 and face mask use highlight some important health literacy issues. Good levels of health literacy are crucial because the effective prevention of communicable diseases requires individuals to understand and take personal responsibility to avoid behaviors that present a high risk for infection and understand the rationale behind recommendations calling for social responsibility to fight the pandemic (39, 40).

The rate of face mask re-use in this sample was 54%, where 83.8% of these participants reused each mask 1–2 times. This relatively high rate of facemask re-use in a fairly wealthy sample may be explained by an actual or perceived lack of mask stocks during the survey period. It is clear that a stable supply of quality face masks is required to achieve large-scale mass masking within a population (41), however, during the time of data collection regional studies and local news reports indicated that the market was flooded with fake face masks, the price of masks escalated, and there were occasional shortages (42). In consequence, the practice of reusing face masks was also prevalent, as detailed in some local studies and news reports (43). These circumstances seemed to have contributed to a high level of stress in the general public, a recent study also showed worsening sleep quality (30–40%) and causing insomnia (30%) among the general public (44). These studies seem to support our findings on high rate of mask reuse and the potential of this to be associated with depressive symptoms in Hong Kong. Unfortunately, nearly 70% of respondents felt unsafe to reuse face masks and almost 80% stated that they were unclear about guidelines for reuse. This lack of clarity combined with a high level of perceived susceptibility to COVID-19 infection is very likely to cause additional mental distress in the general public. To some extent this lack of health literacy is understandable given the huge amounts of conflicting COVID-19 information available, which has recently been described as an “infodemic” (45). This “infodemic” may be particularly problematic for people who have difficulty locating and processing health advice, such as those experiencing depression.

The results also show that a higher proportion of people with probable depression were unclear about the reuse guidelines and tended to wear face masks for self-protection more often when compared with those with low levels of depressive symptoms. Whereas, participants who had better knowledge of the disease pandemic and higher perceived self-efficacy to wear masks properly were less likely to report depressive symptoms. These results seem to suggest that there is an important relationship between COVID-19 health literacy and depressive symptoms, a finding that is supported by the results of a recent Vietnamese study showing that a one score increment increase of COVID-19 health literacy resulted in 5% lower likelihood of having probable depression (46). Although these studies cannot demonstrate cause and effect, and there is a potential bi-directional relationship between health literacy and depression, these results have potential implications for health literacy provision during communicable disease epidemics. For example, this may suggest that improving health literacy may help to reduce depressive symptoms, or alternatively that COVID-19 health literacy is poorly grasped by people with depressive symptoms and therefore a tailored approach is required to improve the clarity of health literacy information provided for this group.

Our findings also indicate that participants who believed themselves to be more susceptible to the disease and perceived high severity of the disease outbreak were most likely to report probable depression. In addition, the significant predictors identified in the final regression model accounted for 16% of the overall variance in levels of depressive symptoms indicating probable depression. The addition of the HBM variables in model 3 resulted in explaining an additional 9% of the variance in depression, highlighting that these beliefs/attitudes account for greater variance than demographics and face mask use practice/beliefs combined. This finding may indicate that modifying COVID-19 related health beliefs could be a useful target for interventions to reduce depressive symptoms associated with COVID-19. In accordance with our initial hypotheses, it is possible that participants had higher levels of depressive symptoms because they felt distressed and overwhelmed by the threats posed by COVID-19 or conversely that the presence of depression/anxiety may magnify an individual's perceptions of the severity of the disease and their likelihood of contracting it. Indeed, it is well-established that people with depressive symptoms have a tendency to expect negative outcomes and can become preoccupied with negative thoughts, which are likely to both maintain and exacerbate levels of depressive symptoms (14). Irrespective of the reasons for these findings, our results seem to suggest that public health information about COVID-19 should be concise and aim to target peoples' COVID-19 health beliefs that may be a source of distress and improve their perception of self-efficacy to protect themselves from becoming infected.

## Study Limitations

This study has some methodological limitations that require consideration. This was an online survey utilizing a convenience sampling approach; therefore, the participants are unlikely to be representative of the general Hong Kong population and this severely limits the generalizability of the study findings. For example, all respondents were able to use/access the internet, females were over-represented in the sample and we found some significant differences in demographic characteristics across genders. Also, we did not ask respondents to specify their ethnic group, and given the online mode of the survey we are unable to be certain that all respondents were from Hong Kong or verify their age/other demographic characteristics, further limiting the potential generalisability of the findings. The use of a non-probability sample in the current study also introduces potential bias resulting from selectively recruiting participants who may be more distressed by the pandemic, which may explain the high prevalence of probable depression. The HBM items were newly constructed with brief evaluation of psychometric properties which may compromise the measurement quality. Nonetheless, the use of FMUS and PHQ-9 is a study strength as they were validated with good psychometric properties (23, 28, 29). Recently, some published studies have adopted one or two items for measuring face mask use practice without comprehensive evaluation on psychometric properties (47). Therefore, future studies should adopt the validated instruments like FMUS and PHQ-9 for evaluation of the phenomenon.

## Conclusions

The high point-prevalence of probable depression and suicidal ideation during COVID-19 in Hong Kong is very concerning and seems to have increased since late 2019. However, our estimate of the prevalence of probable depression in the current study should be viewed with caution due to the convenience sampling method employed, therefore future studies should recruit a representative probability sample in order to draw more reliable conclusions. People who perceived that they are at greater risk from the virus, who engage in higher levels of unsafe face mask use and who are unclear about COVID-19 related health information are more likely to report symptoms indicative of probable depression. These findings may suggest that more emphasis should be placed on improving the clarity, quality and accessibility of COVID-19 related information to improve overall health literacy. This information could be specifically tailored toward modifying COVID-19 related health beliefs in people who feel highly distressed by the pandemic.

## Data Availability Statement

The raw data supporting the conclusions of this article will be made available by the authors, without undue reservation.

## Ethics Statement

The studies involving human participants were reviewed and approved the Human Subjects Ethics Sub-committee of the Hong Kong Polytechnic University (reference no: HSEARS20200227002-01). The patients/participants provided their written informed consent to participate in this study.

## Author Contributions

SL: conception and design of the study and acquisition of data. SL and TC: data analysis. SL, DB, TC, LS, and TF: interpretation of data. DB and TC: drafting the manuscript. Y-TX, HH, and LS: critically review. All authors contributed to the article and approved the submitted version.

## Conflict of Interest

The authors declare that the research was conducted in the absence of any commercial or financial relationships that could be construed as a potential conflict of interest.

## References

[1] Sinyor M Rezmovitz J Zaretsky A. Screen all for depression. BMJ. (2016) 2016:352. 10.1136/bmj.i1617 27026236

[2] World Health Organization The World Health Report 2001: Mental health: new understanding, new hope: World Health Organization (2001).

[3] Lim GY Tam WW Lu Y Ho CS Zhang MW Ho RC. Prevalence of depression in the community from 30 countries between 1994 and 2014. Sci Rep. (2018) 8:2861. 10.1038/s41598-018-21243-x 29434331 PMC5809481

[4] Rajkumar RP COVID-19 and mental health: a review of the existing literature. Asian J Psychiatry. (2020) 2020:102066 10.1016/j.ajp.2020.102066 PMC7151415 32302935

[5] Torales J O'Higgins M Castaldelli-Maia JM Ventriglio A. The outbreak of COVID-19 coronavirus and its impact on global mental health. Int J Soc Psychiatry. (2020) 2020:0020764020915212. 10.1177/0020764020915212 32233719

[6] Wang Y Di Y Ye J Wei W. Study on the public psychological states and its related factors during the outbreak of coronavirus disease 2019 (COVID-19) in some regions of China. Psychol Health Med. (2020) 2020:1–10. 10.1080/13548506.2020.1746817 32223317

[7] Gunnell D Appleby L Arensman E Hawton K John A Kapur N. Suicide risk and prevention during the COVID-19 pandemic. Lancet Psychiatry. (2020) 7:468–71. 10.1016/S2215-0366(20)30171-1 32330430 PMC7173821

[8] Zhao N Huang Y Chinese mental health burden during COVID-19 outbreak: a web-based cross-sectional survey. Asian J Psychiatry. (2020) 51:102052 10.1016/j.ajp.2020.102052 PMC7195325 32361387

[9] Zhang J Lu H Zeng H Zhang S Du Q Jiang T. The differential psychological distress of populations affected by the COVID-19 pandemic. Brain Behav Immun. (2020) 87:49–50. 10.1016/j.bbi.2020.04.031 32304883 PMC7156946

[10] Chinese University of Hong Kong SoPH CU Medicine Announces the Community Response Study Results During the Early Phase Of The COVID-19 Outbreak in Hong Kong. CUHK Communications and Public Relations Office (2020). Available online at: https://www.cpr.cuhk.edu.hk/en/press_detail.php?id=3234&t=cu-medicine-announces-the-community-response-study-results-during-the-early-phase-of-the-covid-19-outbreak-in-hong-kong (accessed October 07, 2020).

[11] Qiu J Shen B Zhao M Wang Z Xie B Xu Y. A nationwide survey of psychological distress among Chinese people in the COVID-19 epidemic: implications and policy recommendations. General Psychiatry. (2020) 33:e100213. 10.1136/gpsych-2020-100213 32215365 PMC7061893

[12] Egede LE Ellis C. The effects of depression on diabetes knowledge, diabetes self-management, and perceived control in indigent patients with type 2 diabetes. Diabet Technol Ther. (2008) 10:213–9. 10.1089/dia.2007.0278 18473696

[13] Al-Amer R Ramjan L Glew P Randall S Salamonson Y. Self-efficacy, depression, and self-care activities in adult Jordanians with type 2 diabetes: the role of illness perception. Issues Mental Health Nursing. (2016) 37:744–55. 10.1080/01612840.2016.1208692 27484761

[14] Krämer LV Helmes AW Seelig H Fuchs R Bengel J. Correlates of reduced exercise behaviour in depression: the role of motivational and volitional deficits. Psychol Health. (2014) 29:1206–25. 10.1080/08870446.2014.918978 24785393

[15] Center for Health Protection. Health Advice (2020). Available online at: https://www.coronavirus.gov.hk/eng/health-advice.html (accessed October 07, 2020).

[16] World Health Organization Coronavirus Disease (COVID-19) Advice for the Public: When and How to Use Masks. (2020). Available online at: https://www.who.int/emergencies/diseases/novel-coronavirus-2019/advice-for-public/when-and-how-to-use-masks (accessed October 07, 2020).

[17] Cowling BJ Ali ST Ng TW Tsang TK Li JC Fong MW. Impact assessment of non-pharmaceutical interventions against coronavirus disease 2019 and influenza in Hong Kong: an observational study. Lancet Public Health. (2020) 5:e279–88. 10.1016/S2468-2667(20)30090-6 32311320 PMC7164922

[18] Stallones L Beseler C. Safety practices and depression among farm residents. Ann Epidemiol. (2004) 14:571–8. 10.1016/j.annepidem.2003.11.004 15350957

[19] Beseler CL Stallones L. Safety knowledge, safety behaviors, depression, and injuries in Colorado farm residents. Am J Ind Med. (2010) 53:47–54. 10.1002/ajim.20779 19921707

[20] Champion VL Skinner CS The health belief model. Health behavior and health education: Theory, research, and practice. Health Commun. (2008) 4:45–65. 10.1080/10410236.2013.873363

[21] Lam SC Arora T Grey I Suen LKP Huang EYZ Li D Lam KBH. Perceived risk and protection from infection and depressive symptoms among healthcare workers in mainland china and Hong Kong during COVID-19. Front. Psychiatry. (2020) 11:686. 10.3389/fpsyt.2020.00686 32765321 PMC7378321

[22] Ho H. Use of face masks in a primary care outpatient setting in Hong Kong: knowledge, attitudes and practices. Public Health. (2012) 126:1001–6. 10.1016/j.puhe.2012.09.010 23153561 PMC7111693

[23] Lam SC Chong ACY Chung JYS Lam MY Chan LM Shum CY Methodological study on the evaluation of face mask use scale among public adult: cross-language and psychometric testing. Korean J Adult Nurs. (2020) 32:46–56. 10.7475/kjan.2020.32.1.46

[24] Tang CSK Wong CY. Factors influencing the wearing of facemasks to prevent the severe acute respiratory syndrome among adult Chinese in Hong Kong. Prev Med. (2004) 39:1187–93. 10.1016/j.ypmed.2004.04.032 15539054 PMC7133369

[25] Wong CY Tang CSK. Practice of habitual and volitional health behaviors to prevent severe acute respiratory syndrome among Chinese adolescents in Hong Kong. J Adolescent Health. (2005) 36:193–200. 10.1016/j.jadohealth.2004.02.024 15737774 PMC7129542

[26] Sim SW Moey KSP Tan NC. The use of facemasks to prevent respiratory infection: a literature review in the context of the health belief model. Singapore Med J. (2014) 55:160. 10.11622/smedj.2014037 24664384 PMC4293989

[27] Brislin RW Back-translation for cross-cultural research. J Cross Cult Psychol. (1970) 1:185–216. 10.1177/135910457000100301

[28] Wang W Bian Q Zhao Y Li X Wang W Du J. Reliability and validity of the Chinese version of the Patient Health Questionnaire (PHQ-9) in the general population. Gen Hospital Psychiatry. (2014) 36:539–44. 10.1016/j.genhosppsych.2014.05.021 25023953

[29] Kroenke K Spitzer RL Williams JB. The PHQ-9: validity of a brief depression severity measure. J Gen Intern Med. (2001) 16:606–13. 10.1046/j.1525-1497.2001.016009606.x 11556941 PMC1495268

[30] Ni MY Yao XI Leung KS Yau C Leung CM Lun P. Depression and post-traumatic stress during major social unrest in Hong Kong: a 10-year prospective cohort study. Lancet. (2020) 395: P273–84. 10.1016/S0140-6736(19)33160-5 31928765

[31] Yu X Tam WW Wong PT Lam TH Stewart SM. The patient health questionnaire-9 for measuring depressive symptoms among the general population in Hong Kong. Comprehens Psychiatry. (2012) 53:95–102. 10.1016/j.comppsych.2010.11.002 21193179

[32] Chin WY Chan KT Lam CL Wong SY Fong DY Lo YY. Detection and management of depression in adult primary care patients in Hong Kong: a cross-sectional survey conducted by a primary care practice-based research network. BMC Family Practice. (2014) 15:30. 10.1186/1471-2296-15-30 24521526 PMC3937039

[33] Huang Y Zhao N. Generalized anxiety disorder, depressive symptoms and sleep quality during COVID-19 outbreak in China: a web-based cross-sectional survey. Psychiatry Res. (2020) 288:112954. 10.1016/j.psychres.2020.112954 32325383 PMC7152913

[34] Moriarty AS Gilbody S McMillan D Manea L. Screening and case finding for major depressive disorder using the patient health questionnaire (PHQ-9): a meta-analysis. Gen Hosp Psychiatry. (2015) 37:567–76. 10.1016/j.genhosppsych.2015.06.012 26195347

[35] Pilling S Anderson I Goldberg D Meader N Taylor C. Depression in adults, including those with a chronic physical health problem: summary of NICE guidance. BMJ. (2009) 339:b4108. 10.1136/bmj.b4108 19861376 PMC3230232

[36] Hospital Authority Legislative Council Q11: Annex 1 to 3. (2020). Available online at: https://gia.info.gov.hk/general/201105/18/P201105180167_0167_79006.pdf (accessed October 07, 2020).

[37] Census Statistics Department HKSAR Population Estimates: Population by Age Group and Sex. (2020). Available online at: https://www.censtatd.gov.hk/hkstat/sub/sp150.jsp?tableID=002&ID=0&productType=8 (accessed October 07, 2020).

[38] Maunder R Hunter J Vincent L Bennett J Peladeau N Leszcz M. The immediate psychological and occupational impact of the 2003. SARS outbreak in a teaching hospital. CMAJ: Can Med Assoc J. (2003) 168:1245–51. 12743065 PMC154178

[39] Van den Hoven M Why one should do one's bit: thinking about free riding in the context of public health ethics. Public Health Ethics. (2012) 5:154–60. 10.1093/phe/phs023

[40] Paakkari L Okan O. COVID-19: health literacy is an underestimated problem. Lancet Public Health. (2020) 5:e249–50. 10.1016/S2468-2667(20)30086-4 32302535 PMC7156243

[41] Greenhalgh T Schmid MB Czypionka T Bassler D Gruer L. Face masks for the public during the covid-19 crisis. BMJ. (2020) 369:m1435. 10.1136/bmj.m1435 32273267

[42] Lam SC Suen LKP Cheung TCC. Global risk to the community and clinical setting: flocking of fake masks and protective gears during the COVID-19 pandemic. Am J Infect Control. (2020). 10.1016/j.ajic.2020.05.008 32405127 PMC7219383

[43] Tam VC Tam SY Poon WK Law HKW Lee SW. A reality check on the use of face masks during the COVID-19 outbreak in Hong Kong. EClinicalMedicine. (2020) 22:100356. 10.1016/j.eclinm.2020.100356 32337502 PMC7180351

[44] Yu BY-M Yeung W-F Lam JC-S Yuen SC-S Lam SC Chung VC-H. Prevalence of sleep disturbances during covid-19 outbreak in an urban Chinese population: a cross-sectional study. Sleep Med. (2020) 74:18–24. 10.1016/j.sleep.2020.07.009 32836181 PMC7367777

[45] Zarocostas J. How to fight an infodemic. Lancet. (2020) 395:676. 10.1016/S0140-6736(20)30461-X 32113495 PMC7133615

[46] Nguyen HC Nguyen MH Do BN Tran CQ Nguyen TT Pham KM. People with suspected COVID-19 symptoms were more likely depressed and had lower health-related quality of life: the potential benefit of health literacy. J Clin Med. (2020) 9:965. 10.3390/jcm9040965 32244415 PMC7231234

[47] Chen X Ran L Liu Q Hu Q Du X Tan X. hand hygiene, mask-wearing behaviors and its associated factors during the COVID-19 epidemic: a cross-sectional study among primary school students in Wuhan, China. Int J Environ Res Public Health. (2020) 17:2893. 10.3390/ijerph17082893 32331344 PMC7215913

