# Supplementary material for: Association Between Depression, Health Beliefs, and Face Mask Use During the COVID-19 Pandemic
Source: Front Psychiatry. 2020 Oct 22;11:571179. doi: 10.3389/fpsyt.2020.571179 (PMC7642487; doi:10.3389/fpsyt.2020.571179)
Supplement: Supplementary file 1 [file Data_Sheet_1.docx]

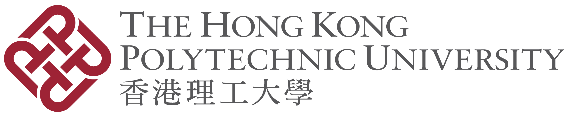
Part 1: General Background

1a. In which country are you living now?

1b. In what city/town/region are you living now (i.e. London, Rio, New York, etc.)

1a and 1b is for the need of identification of areas being investigated.

1. Age：_____
2. What is your identifying gender? ❑ Male ❑ Female ❑Other
3. Number of family member(s)/person(s) living under the same roof (excluding yourself, 0 = living alone)：_____
4. What is the highest degree or level of school you have completed? ❑ Elementary or below ❑ High school, diploma or equivalent received ❑ University degree (Associate or Bachelor) ❑ Post-graduate degree (Master or PhD/Doctorate)
5. Marital status：❑ Single ❑ In a relationship ❑ Married ❑ Divorced/Separated ❑ Windowed
6. Have you previously or are you currently working in the health care industry?

❑ Yes🡪❑Doctor ❑Nurse ❑Allied healthcare professionals ❑Patient supporting staff (E.g.: Health care assistant/Clerical staff/Janitorial service/Delivery service)

❑No, I have not worked nor am currently working in the health care industry

1. Do you come into contact or have close contact with patients in your current occupation?

❑ Never ❑ Rarely ❑ At least once a month ❑ At least once a week ❑ At least once a day

1. Monthly income (USD)：❑ <1,280 ❑1,281－2,560 ❑ 2,561－3,850 ❑3,851－5,130 ❑5,131－7,700 ❑>7,701
2. How many times in the PAST YEAR have you experienced influenza-like symptoms (i.e. cough, fever, sore throat, runny nose, etc.)? ______ times (Please express in number; 0 = no such experience)

Part 2: The Practice of Using Face Mask

(By recalling the practice of using face mask in the past two weeks, please select the best answer to describe your actual situation. There is no right or wrong answer.)

|  | Never | Rarely | Sometimes | Frequently | Always |
| --- | --- | --- | --- | --- | --- |
| 1. I wear a facial mask in public venues to protect myself against influenza like illnesses | ❑ | ❑ | ❑ | ❑ | ❑ |
| 1. I wear a facial mask in doctor’s clinic to protect myself against influenza like illnesses | ❑ | ❑ | ❑ | ❑ | ❑ |
| 1. I wear a facial mask at home when I have symptoms of influenza like illnesses | ❑ | ❑ | ❑ | ❑ | ❑ |
| 1. I wear a facial mask in public venues when I have symptoms of influenza like illnesses | ❑ | ❑ | ❑ | ❑ | ❑ |
| 1. I wear a facial mask in doctor’s clinic when I have symptoms of influenza like illnesses | ❑ | ❑ | ❑ | ❑ | ❑ |
| 1. I wear a facial mask at home when family members have influenza like illnesses | ❑ | ❑ | ❑ | ❑ | ❑ |

| 1. During the outbreak of disease, what type of mask do you currently wear in public venues?   ❑ Paper mask or gauze mask ❑ Washable sponge or cotton mask ❑ Surgical mask ❑ Activated carbon mask ❑ N95 respirator  ❑ Others:___________ | 1. During the outbreak of disease, what type of mask do you want to wear the most in public venues?   ❑ Paper mask or gauze mask ❑ Washable sponge or cotton mask ❑ Surgical mask ❑ Activated carbon mask ❑ N95 respirator  ❑ Others:___________ |
| --- | --- |
| 1. During the outbreak of disease, how often do you reuse the face mask?   ❑Never reuse  ❑Rarely（1-2 times）  ❑Sometimes（3-4 times）  ❑Frequently（5-6 times）  ❑Always（7 times or more） | 1. What kind(s) of method do you choose to disinfect the used face mask? (available for multiple answers)   ❑Never reuse  ❑Reuse without disinfection  ❑Sunlight  ❑Ultraviolet light  ❑Alcohol  ❑Steam  ❑Dry heat（i.e. hot air / oven）  ❑Others:__________________________ |
| 1. Referring to question 13, do you think the face mask is safe to reuse?   ❑Very unsafe  ❑Unsafe  ❑Unsure  ❑Safe  ❑Very safe | 1. Do you think the guideline of reuse of face mask is clear now?   ❑very unclear  ❑unclear  ❑clear  ❑very clear |

Part 3：The Reason of Using Face Mask

|  | Not at all | Slightly | Very | Extremely |
| --- | --- | --- | --- | --- |
| 1. Do you feel vulnerable to contracting the disease of outbreak? | ❑ | ❑ | ❑ | ❑ |
|  | Yes | | No | |
| 1. Whether you know or have close contact with any individuals infected with the disease of outbreak? | ❑ | | ❑ | |
| 1. Whether you have symptoms similar to disease of outbreak (such as sore throat, cough, fever, muscle ache, and shortness of breath)? | ❑ | | ❑ | |
|  | Not at all | Slightly | Very | Extremely |
| 1. What is the degree to which you are fearful of contracting the disease of outbreak? | ❑ | ❑ | ❑ | ❑ |
| 1. What is the degree to which you are worried that your city/town/region may become quarantined due to the widespread disease of outbreak? | ❑ | ❑ | ❑ | ❑ |
| 1. What is the degree to which you agree wearing facemasks could prevent contracting and spreading the disease of outbreak? | ❑ | ❑ | ❑ | ❑ |
| 1. What is the degree to which you have difficulty in obtaining facemasks? | ❑ | ❑ | ❑ | ❑ |
| 1. What is the level of discomfort when you are wearing face mask? | ❑ | ❑ | ❑ | ❑ |
| 1. What is the degree to which the local government encourages you to wear facemask? | ❑ | ❑ | ❑ | ❑ |
| 1. What is the degree to which your family members and/or peers encourage you to wear face masks? | ❑ | ❑ | ❑ | ❑ |
| 1. Do you perceive you have adequate knowledge about the disease of outbreak? | ❑ | ❑ | ❑ | ❑ |
| 1. Do you think local health authorities have provided adequate information on the disease of outbreak? | ❑ | ❑ | ❑ | ❑ |
| 1. What is the degree to which you believe you are able to properly wear mask? | ❑ | ❑ | ❑ | ❑ |

Part 4：Mental Health Questionnaire

| Over the last 2 weeks, how often have you been bothered by any of the following problems? | Not at all | Several days | More than half the days | Nearly every day |
| --- | --- | --- | --- | --- |
| 1. Little interest or pleasure in doing things | ❑ | ❑ | ❑ | ❑ |
| 1. Feeling down, depressed, or hopeless | ❑ | ❑ | ❑ | ❑ |
| 1. Trouble falling or staying asleep, or sleeping too much | ❑ | ❑ | ❑ | ❑ |
| 1. Feeling tired or having little energy | ❑ | ❑ | ❑ | ❑ |
| 1. Poor appetite or overeating | ❑ | ❑ | ❑ | ❑ |
| 1. Feeling bad about yourself or that you are a failure or have let yourself or your family down | ❑ | ❑ | ❑ | ❑ |
| 1. Trouble concentrating on things, such as reading the newspaper or watching television | ❑ | ❑ | ❑ | ❑ |
| 1. Moving or speaking so slowly that other people could have noticed. Or the opposite being so fidgety or restless that you have been moving around a lot more than usual | ❑ | ❑ | ❑ | ❑ |
| 1. Thoughts that you would be better off dead, or of hurting yourself | ❑ | ❑ | ❑ | ❑ |

END
