# Supplementary material for: Association Between Depression, Health Beliefs, and Face Mask Use During the COVID-19 Pandemic
Source: Front Psychiatry. 2020 Oct 22;11:571179. doi: 10.3389/fpsyt.2020.571179 (PMC7642487; doi:10.3389/fpsyt.2020.571179)
Supplement: Supplementary file 2 [file Data_Sheet_2.docx]

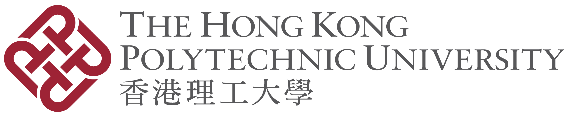
第一部份：背景資料

1. 年齡：_____
2. 性別：❑ 男 ❑ 女
3. 同住家庭成員人數：_____ 人
4. 教育程度：❑小學或以下 ❑中學 ❑ 大專或以上
5. 婚姻狀況：❑單身 ❑交往中 ❑已婚 ❑ 離婚/分居 ❑喪偶
6. 職業：曾經/現在從事醫護行業？

❑ 是🡪❑醫生 ❑護士 ❑專職醫療 ❑病人服務助理職系(例如：抽血員/健康助理/文員/清潔員/運送員)

❑ 否

1. 您的工作會接觸病人嗎？ ❑從不/不適用 ❑很少 ❑至少每月1次 ❑至少每週1次 ❑至少每天1次
2. 每月收入：❑ ≤9,999元 ❑10,000－19,999元 ❑ 20,000－29,999元
    ❑30,000－39,999元 ❑40,000－59,999元 ❑≥60,000元
3. 過去一年內，您曾經有多少次出現類似呼吸道感染（例如：咳嗽、發燒、喉痛等）的病徵？

______ 次 (請以數字表示，例子：0 = 沒有)

第二部份：使用口罩的情況

(請您回想過去兩星期使用口罩的情況。請注意:本問卷的答案無分對錯，只需根據自己的實際情況填寫即可。)

| 1. 疫情期間，您現時在公眾場所，配戴那類口罩？   ❑一般紙口罩或紗布口罩 ❑可清洗海綿或綿布口罩 ❑外科口罩 ❑活性碳口罩 ❑N95 口罩  ❑其他:___________ | 1. 疫情期間，您現時在公眾場所，最想配戴那類口罩？   ❑一般紙口罩或紗布口罩 ❑可清洗海綿或綿布口罩 ❑外科口罩 ❑活性碳口罩 ❑N95 口罩  ❑其他:___________ |
| --- | --- |
| 1. 疫情期間，您的口罩一般會重複使用多少次？   ❑不重複使用  ❑偶爾重複使用（1-2次）  ❑有時重複使用（3-4次）  ❑時常重複使用（5-6次）  ❑常常重複使用（7次或以上） | 1. 您使用哪種方法去消毒已使用的口罩? (多選題)   ❑不消毒直接重複使用  ❑陽光消毒法  ❑紫外線消毒法  ❑酒精消毒法  ❑蒸煮消毒法  ❑干熱消毒法（例如：熱風/烤箱）  ❑其他 :__________________________ |
| 1. 承上題，您覺得重用的安全性如何？   ❑非常不安全  ❑不安全  ❑不知道  ❑安全  ❑非常安全 | 1. 您認為現時有關口罩重複使用的指引清晰嗎?   ❑非常不清晰  ❑不清晰  ❑清晰  ❑非常清晰 |

|  | 從來沒有 | 不常有 | 有時有 | 時常有 | 常常都有 |
| --- | --- | --- | --- | --- | --- |
| 2.1.我會在公眾場所戴口罩去保護自己免受呼吸道感染 | ❑ | ❑ | ❑ | ❑ | ❑ |
| 2.2.我會在診所內戴口罩去保護自己免受呼吸道感染 | ❑ | ❑ | ❑ | ❑ | ❑ |
| 2.3.當我有類似呼吸道感染的病徵時，我會在家裡戴口罩以防止我的家人受到感染 | ❑ | ❑ | ❑ | ❑ | ❑ |
| 2.4.當我有類似呼吸道感染的病徵時，我會在公共場所戴口罩 | ❑ | ❑ | ❑ | ❑ | ❑ |
| 2.5.當我有類似呼吸道感染的病徵時，我會在診所內戴口罩 | ❑ | ❑ | ❑ | ❑ | ❑ |
| 2.6.當我的家人有類似呼吸道感染的病徵時，我會在家裡戴口罩 | ❑ | ❑ | ❑ | ❑ | ❑ |

第三部份：使用口罩的原因

|  | 從不 | 少許 | 很多 | 非常 |
| --- | --- | --- | --- | --- |
| 3.1.您認為自己容易受到疫症感染嗎？ | ❑ | ❑ | ❑ | ❑ |
|  | 有 | | 沒有 | |
| 3.2.您認識/曾經接觸過感染疫症的人士嗎？ | ❑ | | ❑ | |
| 3.3.您最近有類似疫症感染的病徵嗎？（例如：喉痛、咳嗽、發燒、肌肉痛、呼吸困難等） | ❑ | | ❑ | |

|  | 從不 | 少許 | 很多 | 非常 |
| --- | --- | --- | --- | --- |
| 3.4.您害怕感染疫症的程度為多少? | ❑ | ❑ | ❑ | ❑ |
| 3.5.由於現時疫症於社區廣泛傳播，您擔心現時的居住地將會變成疫區的程度為多少? | ❑ | ❑ | ❑ | ❑ |
| 3.6.您同意戴口罩可以預防感染及避免傳播疫症的程度為多少? | ❑ | ❑ | ❑ | ❑ |
| 3.7.您獲得口罩的困難的程度為多少？ | ❑ | ❑ | ❑ | ❑ |
| 3.8.您在配戴口罩時感到不適的程度為多少? | ❑ | ❑ | ❑ | ❑ |
| 3.9.您認為本地政府鼓勵您配戴口罩的程度為多少? | ❑ | ❑ | ❑ | ❑ |
| 3.10.您認為家人及/或同儕鼓勵您配戴口罩的程度為多少? | ❑ | ❑ | ❑ | ❑ |
| 3.11.您是否認為自己有足夠關於疫症的知識？ | ❑ | ❑ | ❑ | ❑ |
| 3.12.您認為本地健康衛生部門是否提供了足夠的疫症知識？ | ❑ | ❑ | ❑ | ❑ |
| 3.13.您認為您可以正確配戴口罩的程度為多少? | ❑ | ❑ | ❑ | ❑ |

第四部份：心理健康調查

| 在過去兩個星期，有多少時候您受到以下任何問題所困擾？ | 完全沒有 | 幾天 | 一半以上的天數 | 幾乎每天 |
| --- | --- | --- | --- | --- |
| 4.1.做事時提不起勁或沒有樂趣 | ❑ | ❑ | ❑ | ❑ |
| 4.2.感到心情低落、沮喪或絕望 | ❑ | ❑ | ❑ | ❑ |
| 4.3.入睡困難、睡不安穩或睡眠過多 | ❑ | ❑ | ❑ | ❑ |
| 4.4.感覺疲倦或沒有活力 | ❑ | ❑ | ❑ | ❑ |
| 4.5.食慾不振或吃太多 | ❑ | ❑ | ❑ | ❑ |
| 4.6.覺得自己很糟 或 覺得自己很失敗，或讓自己或家人失望 | ❑ | ❑ | ❑ | ❑ |
| 4.7.對事物專注有困難， 例如閱讀報紙或看電視時 | ❑ | ❑ | ❑ | ❑ |
| 4.8.動作或說話速度緩慢到別人已經察覺，或正好相反—煩躁或坐立不安、動來動去的情況更勝於平常 | ❑ | ❑ | ❑ | ❑ |
| 4.9.有不如死掉或用某種方式傷害自己的念頭 | ❑ | ❑ | ❑ | ❑ |

END
